# Chemical Treatment of Some Lignosulfonates Under DBD Plasma Conditions–II: Characterization of the Modified Lignosulfonates Microparticles

**DOI:** 10.3390/polym18141756

**Published:** 2026-07-18

**Authors:** Georgeta Cazacu, Daniela Pamfil, Oana Chirilă, Marian Totolin, Diana Ciolacu, Alina Ghilan, Loredana Niţă, Tudorachi Niţă, Cornelia Vasile

**Affiliations:** 1 Department of Physical Chemistry of Polymers, “Petru Poni” Institute of Macromolecular Chemistry of the Romanian Academy, 41A Grigore Ghica Vodă Alley, 700487 Iasi, Romania; gcazacu@icmpp.ro (G.C.); oana_0206@yahoo.com (O.C.); cvasile@icmpp.ro (C.V.); 2 Department of Electroactive, Polymers and Plasmochemistry, “Petru Poni” Institute of Macromolecular Chemistry of the Romanian Academy, 41A Grigore Ghica Vodă Alley, 700487 Iasi, Romania; mtotolin@yahoo.com; 3 Department of Natural Polymers, Bioactive and Biocompatible Materials, “Petru Poni” Institute of Macromolecular Chemistry of the Romanian Academy, 41A Grigore Ghica Vodă Alley, 700487 Iasi, Romania; dciolacu@icmpp.ro (D.C.); diaconu.alina@icmpp.ro (A.G.); lnazarie@icmpp.ro (L.N.); ntudor@icmpp.ro (T.N.)

**Keywords:** lignosulfonate, plasma discharge, microparticles, surface functionalization, controlled morphologies, thermal properties, antioxidant activity

## Abstract

The chemically modified ammonium lignosulfonate (ALS) powders with carboxylic acids such as, oleic (OA) and lactic acid (LA) and γ-butyrolactone (BL) under dielectric barrier plasma discharge (DBD) have been characterized by average molecular weight and particle size determinations, morphology examination by optical and electronic microscopy (SEM), the study of the thermal properties by thermogravimetry (TG/DTG), differential scanning calorimetry (DSC), differential thermal analysis (DTA) and antioxidant activity tests by DPPH method. The thermal characterization of the modified lignosulfonates reveals their improved thermal stability comparatively with ALS. It has been established that the obtained microparticles are aggregates of particles, covered by modified polymer and exhibit a particular behavior depending on the chemical structure of the used modifier, leading to multifunctional active lignin-based products with better homogeneity. By surface modification, the antioxidant capacity of modified lignosulfonate powders has been maintained.

## 1. Introduction

Lignosulfonates are derived as by-products or waste of the pulp and paper industry from chemical pulping processes, e.g., sulfite pulping or by sulfonation process of the Kraft lignin. The competitiveness of biorefineries based on sugars production also depends on the interest in the valorization of lignin residue that is becoming increasingly essential because it acts as valuable bioactive agent [1,2]. The global production of lignosulfonates exceeds 1.8 million metric tons/year, with calcium, sodium, and magnesium lignosulfonates accounting for over 82% of total consumption of lignosulfonate. The main utilization (approximately 45%) of lignosulfonates is in construction as water-reducing agents and plasticizers. In United States, lignosulfonate is primarily used as cement additives, dust control agents (nearly 35% of admixture formulations lignosulfonate-based chemical additives), and animal feed binders (18%). Advancements in lignosulfonate technology have improved products yield by 24%, supporting the development toward low-emissions. Lignosulfonates market size valued at 684.59 million USD in 2026 and is expected to reach 893.23 million USD by 2035, at a Compound Annual Growth Rate (CAGR) of 3% [3]. Due to the abundance and interesting properties correlating with the presence of the anionic groups, the lignosulfonates’ isolation and use after pulping could be an interesting solution to avoid some environmental problems such as water and soil contamination and more than they could be important candidates for a variety of applications, through chemical and physical modifications. It is already known the use of lignosulfonates as binders in cement, fertilizer in drilling oils, and as dispersants of coal water slurry [4], including improved dispersion and stability of graphene suspensions [5]. In the construction field after a combination of the oxidation–sulphomethylation reactions of sodium lignosulfonate, a concrete superplasticizer with improved properties was obtained [6]. Lignosulfonates-based bioactive and biocompatible coatings as soil improvers bio-catalyzed by laccase and plasticized with glycerol, xylitol, or sorbitol have been obtained. As examples, bio-based bioactive, low CO_2_ impact formulations, plant growth promoter microorganisms or biocatalysts (*Bacillus* species) were added to the coatings to promote soil fertilization. Soil improvers with no toxic effects on the germination and growth process of the model plants, including corn, wheat, tomato, salad [7] have also been developed.

Ammonium lignosulfonates (ALS) are widely used in wood-based panel industries as sustainable bio-based additives, serving both as partial replacements for phenol in phenol–formaldehyde (PF) resins and as direct components in wood adhesives. Due to their high content of phenolic hydroxyl groups, ALS offer high reactivity towards formaldehyde. ALS used to partially substitute phenol (typically below 50% by weight) in lignin-phenol–formaldehyde (LPF) resins are sustainable, water-soluble, bio-based alternatives to petroleum-derived adhesives for wood products, offering an eco-friendly alternative with formaldehyde-scavenging properties [8].

However, the valorization of lignin-based residues is limited by different factors, including poor solubility, carbohydrates contamination, chemical and structural variability. Because the reactivity of the non-modified lignosulfonates could be decreased, they are often modified to increase the reactive sites and improve crosslinking in the adhesive matrices. Incorporating modified ALS can enhance the thermal stability of the resulting resins compared to conventional commercial phenolic resins. ALS as wood adhesives are interesting to obtain products with formaldehyde-free potential and in combination with crosslinkers such as polymeric diphenylmethane diisocyanate or furfuryl alcohol to create eco-friendly, low-toxicity, or fully formaldehyde-free adhesives for particle boards. The inclusion of ammonium lignosulfonate in adhesive formulations (such as with urea–formaldehyde) has been shown to reduce formaldehyde emissions in high-density fiber board because it could act as a formaldehyde scavenger. Therefore, the current tendency is to modify their chemical structure in order to increase their reactivity and to improve some characteristics. Chemical treatments include: methylolation, desmethylolation, phenolation, oxidation (using Fenton’s reagent or laccase), sulfomethylation, chemical grafting, etc. These techniques increase and diversify of the functional groups, improve molecular weight control, and enhance interfacial activity. Therefore, an increasing interest is evident for the incorporation of the chemically modified lignosulfonates (MLS) into multicomponent systems for industrial applications [9,10,11].

DBD plasma-assisted modification offers several advantages over conventional chemical modification methods. It is a dry, environmentally friendly process that requires little or no hazardous chemicals, thereby reducing waste generation and simplifying post-treatment purification. In addition, DBD plasma selectively modifies only the outermost surface layers without affecting the bulk properties of the material. The process enables the introduction of a wide range of functional groups (e.g., hydroxyl, carboxyl) and improves surface wettability, adhesion, and biocompatibility. Furthermore, DBD plasma treatment is rapid, energy-efficient, and can be performed at atmospheric pressure, eliminating the need for expensive vacuum systems and facilitating large-scale industrial implementation.

In the development of novel bio-composites, the modified ammonium lignosulfonates (MAL) are used as binder via the pretreatment of corn straw particles [12] or chemical transformation of lignosulfonates to lignosulfonamides has be applied in order to improve their thermal characteristics [13,14]. Through the redox reaction between aluminum ions and lignin, the phenolic hydroxyl content was increased [15]. They are also used as superplasticizers, adhesive, concrete, filler, extender, binders, etc. [16,17]. Lignosulfonates act as natural antioxidants and antimicrobials due to their phenolic structure, making them valuable in active packaging, cosmetics, and biomedical applications. These scavenge radicals, inhibit bacterial growth, particularly Gram-positive strains, and can be incorporated into polymers to prolong product shelf life.

Microparticles including those containing LS are frequently used in pharmaceutical, food and cosmetic industries due to their hierarchical structures and designable multifunctions [18]. Microparticles show ideal stability, antibacterial effect and biocompatibility. The presence of sulfonate groups in their structure localized both in the surface layer and in the volume has been established in the case of the gel microspheres [19]. Phase separation of polymer–polymer or polymer–surfactant in uniform drops could lead to monodisperse microparticles with various high-order structures [20].

In our previous paper, a new physico-chemical method for modification of lignosulfonates with carboxylic acids as oleic acid, (OA) and lactic acid, (LA) or γ-butyrolactone (BL) under dielectric barrier discharge (DBD) plasma conditions at atmospheric pressure has been developed [21].

The aim of this study is to obtain from ammonium lignosulfonate (ALS) powder by the chemical modifications with carboxylic acids (oleic acid, OA or lactic acid, LA) or butyrolactone (BL), under DBD plasma conditions, new products with improved functionalities (such as ALS-LA, ALS-OA or ALS-BL, respectively), which may be used in manufacturing of biocomposite materials, such functional groups (carboxyl and carbonyl) assuring a good compatibility.

By this method, the ammonium lignosulfonate powder (ALS) reacts with lactic acid (ALS-LA), oleic acid (ALS-OA) or γ-butyrolactone (ALS-BL) respectively and new products as water-soluble modified lignosulfonates (MLS) with improved functionalities, have been obtained. By coupled analysis methods, such as elemental analysis, attenuated total reflectance Fourier transform infrared spectroscopy (ATR-FTIR), near-infrared spectroscopy (NIR) and near-infrared chemical imaging (NIR-CI), as well as nuclear magnetic resonance (^1^H-NMR and ^13^C-NMR), UV–VIS and fluorescence spectroscopies, it has been established that the chemical transformations mainly occurred on the surface of ALS. Moreover, under DBD plasma treatment some polar and oxygen-containing groups (e.g., hydroxyl, carbonyl, carboxyl, ether, etc.) are introduced, thus changing the polar character of the polymer surface and improving the surface properties as it is correlated with some literature data [22].

The data obtained by these techniques indicated that the grafting reaction occurred primarily as a surface phenomenon. The modification degree depended on the type of reagent used. However, the significant changes observed, particularly in the samples treated with lactic acid, indicate that cold plasma also affected the bulk properties. The newly obtained lignosulfonate products possess new functionality capable to increase their compatibility with other polymers, making them promising partner compounds for the manufacturing of composite materials.

The attachment of monomeric chains to the macromolecular network of ammonium lignosulfonate (ALS) was proved by the atomic ratios (C/H, C/O, and O/C) calculated from elemental analysis. Furthermore, the spectroscopic results confirmed that the grafting reaction of ammonium lignosulfonate occurred under cold plasma conditions, leading to the formation of various compounds with special structures and functionalities.

The purpose of the present paper is to evidence the new aspects related to the characteristic properties of the previously obtained interesting MLS microparticles, their morphology (by Scanning Electron Microscopy (SEM) and optical microscopy) and thermal properties (by thermogravimetry (TG), differential scanning calorimetry (DSC) and differential thermal analysis (DTA)). By such complementing spectral data, it has been proved the suitability of the chemically modified lignosulfonates microparticles with controlled morphologies and good thermal properties as partners in multicomponent systems. These aspects were already demonstrated for the use of some lignins as additives in multicomponent polymeric systems [23,24,25] and also their potential applications in biomedicine, as superplasticizers, etc. [26].

For the evaluation and application of some by-products it is an increasing interest and necessity to a detailed knowledge and understanding of their properties and processing conditions that underlined their optima characteristics and activities and how can be utilize them for practical applications.

## 2. Materials and Methods

### 2.1. Materials

Ammonium lignosulfonates (ALS) as dark brown powders are derived from fermented hardwood sulfite liquor (at S.C. “Celohart” S.A., Zarnesti, Romania). Ammonium lignosulfonate is a highly polar, ionic salt which makes it soluble in water, alkaline solutions, methyl alcohol and generally insoluble in common non-polar organic solvents like ether, benzene, and acetone. Its solubility in polar organic solvents (like alcohols or glycerol) is generally low and highly dependent on temperature and the presence of water.

The functional groups analysis of the ALS according to the procedures described in the literature [27] revealed the functional groups contents as follows [21]: methoxyl (9.25%), alcoholic hydroxyl (13.12%), phenolic hydroxyl (12.42%), carbonyl (1.47%) and carboxyl (0.51%). Average molecular mass of the ALS was of Mw = 167,000 (g/mol) and purity > 90%. Studies investigating the modification of the ALS powder using DBD plasma conditions at atmospheric pressure have used oleic acid (OA), lactic acid (LA), and γ-butyrolactone (BL) (from Sigma-Aldrich) having purity higher than 85%. These compounds were selected as reagents to improve functional properties of ALS powder.

Conditions for the ammonium lignosulfonates exposure in dielectric barrier discharge plasma (DBD) have been described in our previously paper [21]. Shortly the working parameters of the DBD plasma are: voltage download <100 kV, electrical current intensity <10^–3^ A, pressure ≤1 atm, power 50 W, frequency 500 Hz and the nitrogen as inert gas. ALS was impregnated with a 5 wt% solution of OA, LA or BL in ethyl ether and deposited on glass slides in plasma reactor, in nitrogen atmosphere, for 30 min. After treatment, the reaction products were extracted with acetone in a Soxhlet extractor for 6 h to remove unreacted reagents and then dried at room temperature.

Modified ammonium lignosulfonates (MLS) with organic acids as lactic (ALS-LA) and oleic (ALS-OA) acids, and γ-butyrolactone (ALS-BL) under dielectric barrier discharge (DBD) plasma at atmospheric pressure are very dark brown powders, high-soluble in water, alkaline solutions, methyl alcohol, and insoluble in usual organic solvents. The chemical attachment of the carboxylic acids and γ-butyrolactone by a grafting reaction onto the surface of the ALS under DBD plasma conditions was proved by the values of atomic ratios (C/H, C/O and O/C), calculated from elemental analysis and confirmed different spectral methods which have been evidenced various special structures and functionalities. In has been concluded that by chemical treatment under DBD plasma discharge some polar and oxygen-containing groups (e.g., hydroxyl, carbonyl, carboxyl, ether, etc.) have been attached to the structure of the ALS, thus changing the polar character of the polymer surface and improving the surface properties in accordance with literature data obtained on various lignocellulosic materials such as tropical hardwoods [22] and organosolv lignin powder (ALCELL) [28].

Trolox (a water-soluble analog of vitamin E) and 2,2-diphenyl-1-picrylhydrazyl (DPPH) stable free radical for evaluation of antioxidant activity have been supplied by Sigma-Aldrich (St. Louis, MO, USA).

In this paper, the microparticles of the chemically modified lignosulfonates have been detailed studied by the determination of their characteristics such as: particle size, molecular weight, Z-average, polydispersity index, etc., the examination of their surface morphologies, and by the determination of their thermal behavior and antioxidant activity by using the following investigation methods.

### 2.2. Investigation Methods

Particle size of polymeric samples was estimated by dynamic light scattering (DLS) technique using a Zetasizer Nano ZS device (Malvern Panalytical Ltd, Worcestershire, UK) with red laser wave length of 633 nm, (in He/N_2_ atmosphere) in conditions previous established [29]. The average molecular weight (Mw) and second virial coefficient (A_2_) of polymeric samples have also been determined by using Zetasizer Nano ZS instrument based on the measurements of static scattered light (SSL) intensity for various concentrations of samples at one angle of 173°. The average molecular weight (Mw) and the second virial coefficient (A_2_) and the size of lignosulfonate particles of the polymer samples were also determined using the same procedure with lignosulfonate particles dispersed at various concentrations, in the range of 0.2–1.0 wt%, at 25 °C. For a more precise determination, the MLS aqueous solutions were sonicated in an ultrasonic water bath, with 4 cycles per minute. The cumulated analysis gives: the apparent hydrodynamic diameter (D_H_) and the polydispersity index (PDI). The system uses a non-invasive back scatter (NIBS) technology, which reduces the multiple scattering effects, where the optics is not in contact with the sample, with back scattered light being detected. During determinations, the Mie method is applied over the whole measuring range from 0.6 nm to 6 μm. DLS studies were performed on aqueous solutions. Thus, the powder samples were dispersed in water, maintaining a constant concentration of 1 wt%. The samples were kept under stirring overnight for complete dispersion and then characterized. For a more precise determination, the MLS aqueous solutions were ultrasonicated in an ultrasonic water bath, with 4 cycles per minute.

In the static light scattering (SLS) model, concentration and agglomeration effects are rigorously controlled by standardized experimental and mathematical protocols from Malvern Panalytical: the Debye/Rayleigh equation based on a series of successive dilutions [30,31]. By graphically representing the equation, solute–solvent interactions were quantified by the second virial coefficient (A_2_). The absolute value of the average molecular weight Mw was determined by mathematical extrapolation to infinite concentration, completely eliminating the concentration dependence from the final calculation. Agglomeration effects were physically eliminated by fine filtration (0.22/0.45). Monodispersity and the absence of large aggregates were validated by dynamic light scattering (DLS) before SLS analysis, ensuring that the optical signal comes exclusively from individual polymer chains.

Scanning Electron Microscopy. The SEM images have been recorded on ESEM Quanta type 200 electronic microscope (FEI Company, Hillsboro, OR, USA) by Environmental Scanning Electron Microscopy (ESEM) combined with Energy Dispersive X-ray Spectroscopy (EDAX/EDX examination mode) which enables high-magnification imaging and elemental analysis of samples in their natural, hydrated, or non-conductive state without needing special preparation like coating. Samples were analyzed in powdered form.

Thermogravimetry (TG/DTG). Thermogravimetric (TG) and derivative thermogravimetric (DTG) curves measurements have been performed on a STA 449 F1 Jupiter device (Netzsch, Selb, Germany). The samples up to 10 mg mass were placed in Al_2_O_3_ crucibles. Samples were heated in the temperature range from 30 to 700 °C at a heating rate of 10 °C min^−1^. Nitrogen was purged for an inert atmosphere at a flow rate of 40 mL min^−1^.

Differential scanning calorimetry (DSC) measurements were conducted on a DSC 200 F3 Maia device (Netzsch, Selb, Germany). A mass of 10 mg of each sample has been heated in pressed and pierced Al_2_O_3_ crucibles at a heating rate of 10 °C min^−1^. Nitrogen was used as inert atmosphere at a flow rate of 50 mL min^−1^.

DPPH Antioxidant Assay. The antioxidant activity was tested on the solutions of the powders. MLS powders have been dissolved in mixtures of the water/EtOH 1/1 ratio with a certain concentration. Volumes of 0.1 mL of each sample were mixed with 4.9 mL of 0.1 mM DPPH ethanolic solution. The mixtures were incubated in dark for 30 min, and then the UV-Vis spectra were recorded using a 10 mm quartz cuvette on a Cary 60 spectrophotometer (Agilent Technologies, Santa Clara, CA, USA) against ethanol as blank. The fading of DPPH solution violet color was quantified based on decreased absorbance at 517 nm, which is directly correlated with the DPPH radical scavenging. The antioxidant potential was expressed as the percentage radical scavenging activity (RSA%) calculated using Equation (1).

The experiments were carried out in triplicate and results of radical scavenging activity were expressed as the mean standard deviation.

RSA% = (A_control_ − A_sample_)/A_control_ × 100 (1)

where A_sample_ is the absorbance of the tested sample and A_control_ is the absorbance of the DPPH solution.

The dependence of RSA% values on sample concentration (2, 4, 6, 8, and 10 mg/mL) was graphically represented to determine IC_50_ values (the concentration of the test sample required for 50% inhibition of radical species). Higher antioxidant activity is associated with a lower IC_50_ value.

Trolox was utilized as a standard antioxidant to compare the antioxidant activity of the investigated samples. The antioxidant value corresponding to Trolox was expressed in units known as Trolox equivalent antioxidant capacity (TEAC) which was calculated as the ratio between IC_50_ of Trolox and IC_50_ of sample. It is worth mentioning that the measurements for both Trolox and the sample were performed under the same conditions. A higher TEAC value indicates a stronger radical scavenging ability.

## 3. Results and Discussions

### 3.1. Average Molecular Weight

The average molecular weight (Mw) of the ALS and MLSs has been determined using aqueous solutions of different concentrations varying between 2 and 10 mg/mL. The results are given in Table 1.

The data of Table 1 indicate that Mw values of modified MLS samples increase after the chemical modification with organic compounds under DBD plasma conditions. The average Mw value of ALS-LA sample shows a slight increase, while the Mw values for ALS-OA and ALS-BL increase about 10 times in respect with the ALS value. In the case of the ALS-OA and ALS-BL samples, reactions occur both in the bulk and on the surface, being predominant at the surface of the ALS particles leading to the increase in the Mw values. In the case of the ASL-LA, competitive reactions occur both in the cleavage of the linkages between the structural units and grafting of the lactic acid chains. These data are in accordance with the results obtained by ATR-FT-IR, ^1^H-NMR and NIR spectroscopy [21]. The second virial coefficient slightly increases for ALS-OA and ALS-BL samples, indicating the interactions with solvent (water) or between particles leading to agglomerates, while the A_2_ value of the ALS-LA decreases; it is likely that some repulsive forces with solvent are present.

### 3.2. Particle Size Analysis

**Figure 1:**
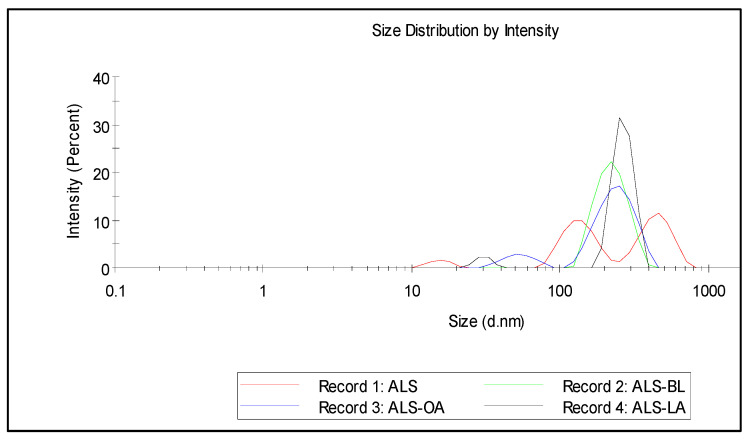
Particle size distribution curves of the ALS and MLS samples.

The particles sizes distribution curves presented in Figure 1 show a high heterogeneity for ALS samples being observed three peaks, while the distribution curves of the physical-chemically modified lignosulfonates (MLS) are bimodal or unimodal, in all cases showing their better homogeneity. The Z values of 0.22–0.45 μm are found for the main peak, while for the second peak values are lower at 0.015–0.14 μm.

The Z-average and polydispersity index (PDI) values evaluated from these curves are given in Table 2. The ALS has two microparticles populations with average diameter of 0.245 and 0.800 μm and PDI is equal to 1.0.

It can be remarked that the Z-average values of the particles diameter and polydispersity (PDI) increase after chemical treatment under DBD plasma exposure. Microparticles of the MLS with relatively uniform diameter have been obtained as they are generally regarded as spherical or irregular particles with diameters ranging from 0.250 to 0.400 μm that could be used in drug delivery and other biomedical applications.

The PDI decreased, indicating that chemical treatment under plasma discharge, generated reaction centers that allowed additional interactions that ultimately led to obtaining an MLS with better homogeneity. It is noteworthy that the ALS-BL sample is the most homogeneous with an average diameter of 0.277 μm and a PDI of 0.69. Solution properties depend on the lignin type and modification applied as it has also been demonstrated for chemically modified organosolv lignin in a previous paper [29].

### 3.3. Scanning Electron Microscopy (SEM)

**Figure 2:**
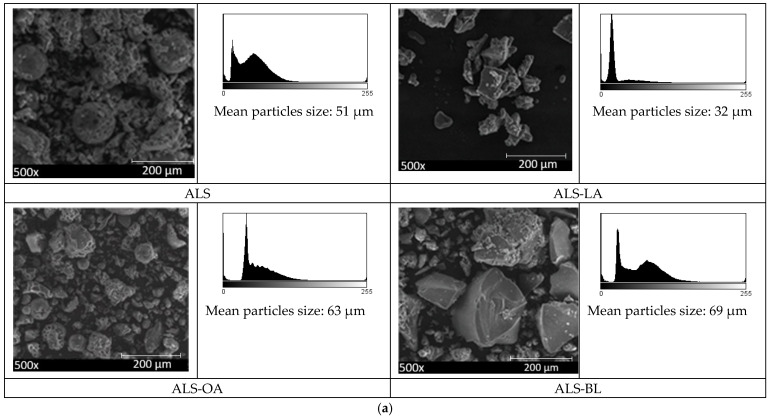

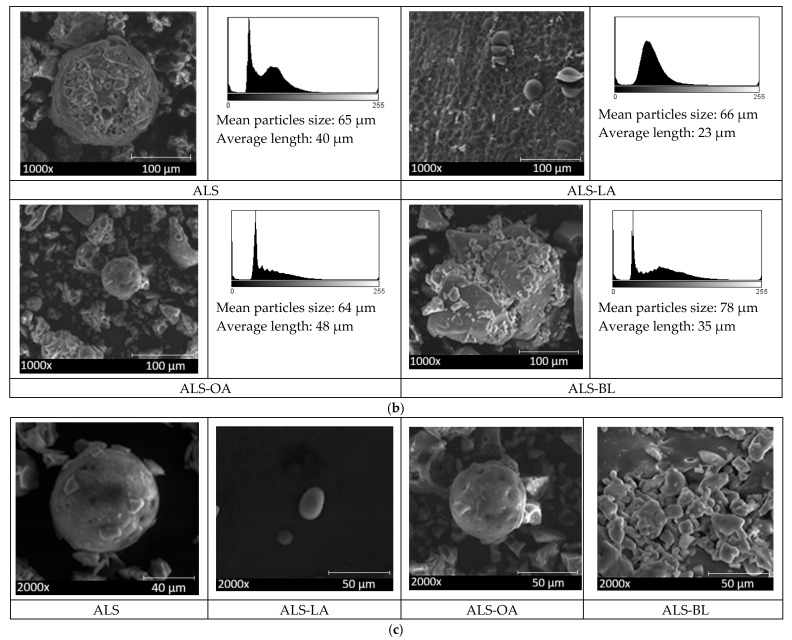
(**a**) SEM images [500×] of the studied lignosulfonate samples. The corresponding particle size distribution histograms are presented alongside each micrograph. The particle size is expressed in μm (x-axis), while the frequency is shown on the *y*-axis. The mean particle size values are indicated for each sample. (**b**) SEM images [1000×] of studied lignosulfonate samples. The corresponding particle size distribution histograms are presented alongside each micrograph. The particle size is expressed in μm (*x*-axis), while the frequency is shown on the *y*-axis. The mean particle size values are indicated for each sample. (**c**) SEM images [2000×] of studied lignosulfonate samples.

The morphology of chemically modified lignosulfonate samples under DBD plasma action has been studied by EDAX/EDX mode. The SEM micrographs at different magnifications of 500×, 1000× are 2000× are displayed in Figure 2a, Figure 2b and Figure 2c, respectively.

A comparison of the surface morphology of ALS and MLS samples indicates that significant morphological changes occur following chemical treatment under DBD plasma conditions. ALS particles exhibit a predominantly spherical morphology, along with a broad particle size distribution. Chemical and plasma-modified samples exhibit improved dispersion suggesting changes in surface properties and intermolecular interactions (in correlation with DLS observations). At higher magnifications, it is observed that the MLS samples exhibit more pronounced surface irregularities. These features likely result from the surface restructuring induced by the introduction of functional groups during chemical treatment under DBD plasma exposure.

The particle size distribution profiles presented in Figure 2a,b are consistent with SEM observations, confirming the presence of a broad size distribution for all samples, some dispersion curves being bimodal. The ALS sample exhibits a broad and asymmetric size distribution, reflecting the coexistence of particles with significantly different sizes (which is consistent with the DLS observations) showing that the sample is the most heterogeneous with a PDI approaching value 1. In contrast, the modified samples exhibit distinct distribution patterns, with some profiles displaying bimodal characteristics (in agreement with the DLS data showing the coexistence of two particle size populations, the difference between the dimensions is explained by the fact that the DLS study is realized in solution and in this case the particles are much less aggregate), which may arise from simultaneous processes, such as particle fragmentation and reorganization during chemical–physical treatment. SEM and particle size distribution analyses provide complementary evidence that chemical treatment under DBD plasma conditions induced both morphological and dimensional changes in the lignosulfonate particles.

The particle sizes obtained by DLS and those observed by SEM highlight the structural behavior of lignosulfonates in different physical states. By DLS, the hydrodynamic diameter was determined in the liquid phase, where the samples show dimensions below 1 micrometer (on the order of hundreds of nanometers), reflecting the conformational changes that occur during DLS modifications. The major difference in size that appears in the case of SEM is due to the fact that this is performed in the dry state, being the result of the secondary agglomeration phenomenon that inevitably occurs during solvent removal (drying), when the solvated primary nanoparticles self-assemble into solid micrometric clusters.

Comparing the surface morphology of ALS with that of the MLS, it can be easily concluded that changes occurred after chemical treatment under DBD plasma conditions. The ALS particles appeared in the spherical form and present a wide size distribution with diameter from 10 μm to 130 μm. The spherical particles are usually associated into the larger compact structure with different sizes.

Lignosulfonates are compounds containing both hydrophobic phenylpropane moieties and strong hydrophilic sulfonic groups are present on the surface of the particles. These polar sulfonic groups may prevent the coverage of the particles by the residual polymeric structures. After chemical modification under DBD plasma, a comparison of the modified ALS with unmodified ALS, evidenced at this process, is less pronounced and the particles can assemble, leading to larger spherical aggregates of lignosulfonate particles, which can be clearly seen in Figure 2c.

The tendency to agglomerate may be caused either by the stronger cohesion forces or due to the H-bonds established between the hydroxyl groups of lignosulfonate derivatives, the molecular chains leading to formation aggregates with different sizes. Also, micrographs obtained for modified products showed the presence of some surfaces of the particles with disaggregated domains containing cavities formed by the release of the lower lignosulfonate particle aggregates. By analyzing micrographs of these aggregates, it can be seen that they exhibited a large specific surface area, which can improve compatibility with other materials. This has been already demonstrated by our previous studies related with the preparation and characterization of complex systems of blends and composites containing unmodified and modified lignosulfonates [23,24,25].

By examination of the surface morphology, it can be seen that the polymeric films display a uniformity from the interior to the surface and they contain fine dispersed lignosulfonate particles. In the case of ALS treated with oleic acid and butyrolactone, the particles seem to be expanded showing that the reaction especially takes place to the particle surface. The ALS-LA samples presented independent particles with smaller dimensions, supposing that the reaction occurred especially in the mass of the particles. These observations are in accordance with the ATR-FTIR and NIR-CI results previously reported [21]. The EDX spectra and microanalysis indicate uniform chemical distribution on the surface of the lignosulfonate particle aggregates. This confirms the hypothesis that under DBD plasma discharge, the reactions took place especially on the surface particles.

### 3.4. Thermal Characterization of Modified Lignosulfonates

The thermal properties of the physically–chemically modified lignosulfonates were studied by thermogravimetry/differential thermal analysis (TG/DTA) and differential scanning calorimetry (DSC).

#### 3.4.1. Thermogravimetry/Differential Thermal Analysis (TG /DTA)

**Figure 3:**
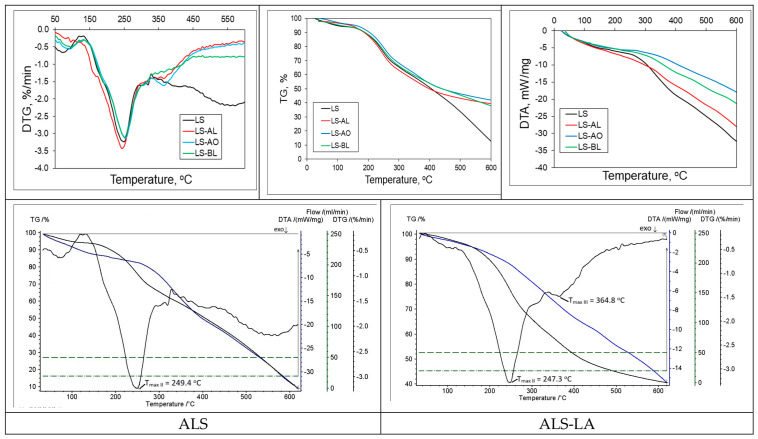

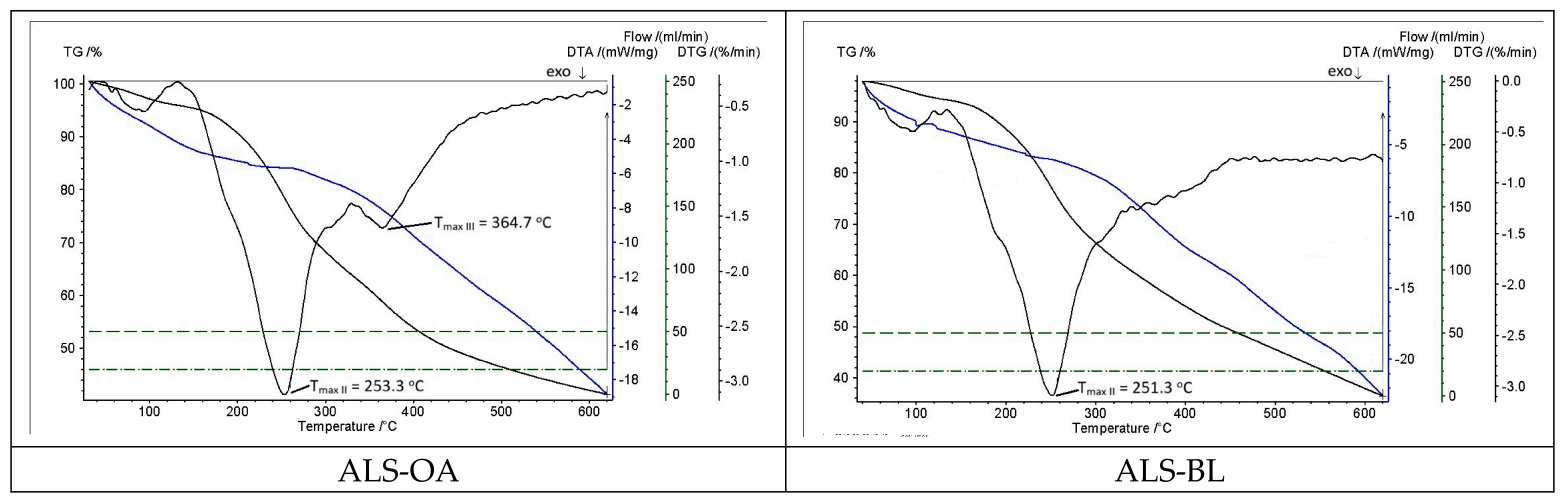
TG/DTG and DTA curves of the unmodified and chemically modified lignosulfonates.

Thermal decomposition of lignin is multi-stages process due to its structural complexity. The lignin thermal decomposition occurs by several competing reactions in the course of which its various bonds are cleaved at different temperatures. The diagrams in Figure 3 and the data in Table 3, are summarized the thermal characteristics of the degradation process for all studied lignosulfonates. Such data are correlated with those obtained by thermogravimetry/mass spectroscopy (TG-MS) studies [32,33]. It has been found that the thermal degradation process of lignin occurs by at least three steps evidenced by three peaks in different temperature ranges and with different maximum decomposition temperatures (rate) at 70–97 °C (peak I), 250 °C (peak II) and 365 °C (peak III), by the release of various types and amounts of volatile products with low molar masses (such as water, formaldehyde, methane, methanol, etc.). Thus, the thermal degradation process of lignin is accompanied by the release of various types and amounts of volatile products with low molar masses (water, formaldehyde, methane, methanol), as it has been shown by thermogravimetry/mass spectroscopy (TG-MS) [32,33]. The decomposition of lignin starts with the cleavage of aliphatic hydroxyl groups (terminal -CH_2_OH groups) leading to water and formaldehyde. Methane and methanol are produced by scission of methoxyl groups. By the lignosulfonate decomposition resulted water, carbon dioxide, carbon monoxide, formaldehyde, methane, methanol and SO_2_ and mercaptans [34]. Brebu et al. [35] showed the formation of ammonia and sulfur dioxide around 250 °C followed to some nitrogen- and sulfur-containing compounds by thermal decomposition of ammonium lignosulfonate.

The studied lignosulfonate products show several decomposition steps. The first step (peak I) at low temperatures ranging from 30 to 100 °C with a mass loss of 2.5–4.4 wt% is assigned to the free and bound water loss (Table 3). From thermogravimetric data it can be observed that the modified lignosulfonates samples (ALS-LA, ALS-OA and ALS-BL) samples lost the water and other volatile compounds at higher temperatures (T_max_ are shifted to high values) in respect to that of ALS, showing an improvement of the thermal stability of the MLS, probably because of the stronger interactions.

In the second degradation step (peak II), which occurred between 100 and 350 °C temperature range, all samples show a similar behavior with a mass loss ranging between 35.8 and 40.7 wt% and a weight loss rate of 3.10–3.28 wt%/min. These losses are likely possible because the gradually breaking of the alkyl-aryl ether bonds, leading to the release of the low mass compounds (probably CO, CO_2_, CH_4_, CH_3_-OH, NH_3_, SO_2_) [35].

The third decomposition step occurs at high temperatures (>350 °C), where it is a possibility of breaking the stronger bonds and release of high molecular weight compounds. In this temperature range, in the DTG curves of the ALS/LA, ALS/OA samples a separate step (peak) is recorded at 364 °C, while in the case of ALS and ALS/BL samples, in the DTG curves a large peak is present. In the case of the modified lignosulfonates, the third thermally decomposition step led to lower mass loss of 59.2–61.4 wt% in comparison with unmodified lignosulfonate (90.3%).

The significant changes took place in the 100–350 °C temperature range, and also at temperatures higher than 350 °C, the process becomes slower, and mass loss differs in these domains, so the TG curve profiles show various steps of weight loss.

The residual mass at 600 °C is higher for modified lignosulfonates (37–42 wt%) than for unmodified one (12.8%), which means that these compounds are more thermally stable. Also, various crosslinking reactions occurred both within the lignosulfonate macromolecules or by the establishing of new bonds between small molecules, during decomposition process, leading to the formation of highly condensed aromatic structures as graphene-like carbon coal [36,37].

It can be concluded that by grafting of the organic compound chains on the ammonium lignosulfonate macromolecule under DBD plasma exposure, the thermal properties are improved due to the molecular interactions involving the incorporated functional groups. The enhanced thermal properties after functionalization under DBD plasma exposure have been obtained for proteins and lipids macromolecules [38].

#### 3.4.2. Differential Scanning Calorimetry

Generally, it is difficult to determine the glass transition temperatures (T_g_) of lignins due to their structural complexity and heterogeneity, molecular weight, crosslinking degree, etc. A typical value for a particular lignin cannot be reported precisely. However, using the differential scanning calorimetry technique, it was found that the T_g_ of lignins ranges from 90 to 180 °C [39,40,41]. Due to the complex structure of ALS, the value of T_g_ was often difficult to measure. From this reason, the T_g_ values for these two samples were determined as half C_p_ extrapolated.

**Figure 4:**
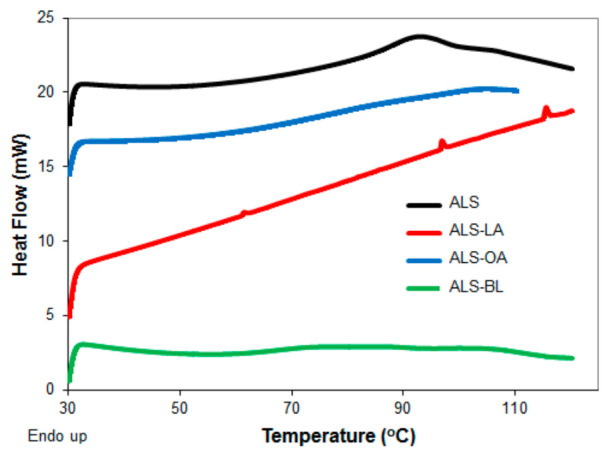
DSC curves of unmodified and modified ammonium lignosulfonate.

In this study, the DSC curves recorded in the first run evidenced the variation in the glass transition of lignosulfonate after chemical treatment with organic compounds under DBD plasma exposure. As it is evidenced in curves in Figure 4, the neat ALS exhibits a broad glass transition and a relatively high temperature (T_g_) at 92.4 °C, due to the aromatic structure and intermolecular H-bonds between functional groups that restrict the molecular mobility. T_g_ of ALS-OA and ALS-BL, observed during first run decreased at approximately 81.7 °C, and 74.3 °C, respectively.

In the DSC curve of the ALS-LA, two glass transitions have been detected at 61.4 and 96.9 °C (Table 4).

### 3.5. DPPH Antioxidant Assay

**Figure 5:**
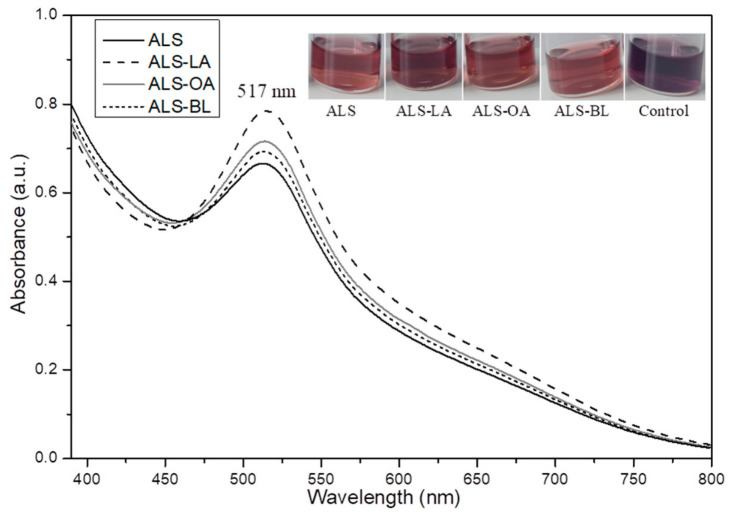
UV-Vis spectra of the ALS and MLS samples at concentration of 10 mg/mL in contact with DPPH solution, both in water/EtOH (1:1 *v*/*v*) mixture.

Due to their phenolic structure, lignosulfonates act as natural antioxidants and antimicrobials (potential antiviral (anti-HIV, anti-HCV) and antifungal effects), making them valuable in active packaging, cosmetics, and biomedical applications [42,43,44]. Based on this statement, the antioxidant activity of MLS through DPPH approach was evaluated. Figure 5 presents the UV-Vis spectra of the ALS and MLS samples, at concentration of 10mg/mL, in water/EtOH (1:1 *v*/*v*) mixture in contact with DPPH solution. Sample blanks were measured and no absorbance attributed to sample was detected at 517 nm, indicating the absence of interference with DPPH assay.

The 517 nm absorption band, typical for DPPH solution, was used for the quantification of radical scavenging activity (RSA %) in reaction with the active lignin-based compounds using Equation (1). A decrease in the DPPH absorption band in reaction with lignosulfonates samples is directly correlated with a free radical scavenging activity. The results are presented in Table 5.

**Figure 6:**
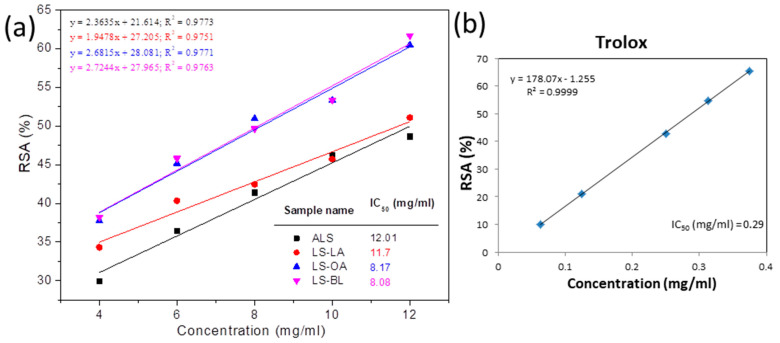
DPPH radical scavenging activity (RSA %) and IC_50_ values of unmodified, modified lignosulfonates (MLS) (**a**), and Trolox (**b**) reference (in water/EtOH (1:1 *v*/*v*) solution).

The relationship between the DPPH radical scavenging activity (RSA %) and the concentration of the samples required to inhibit 50% of the initial DPPH free radicals (IC_50_) are shown in Figure 6. IC_50_ value is inversely related to the antioxidant activity. A high linearity of dependencies (R^2^ > 0.97) is observed, which confirms the applicability of the spectrophotometric approach to quantify antioxidant activity. The antioxidant properties of the lignin’s natural stem result from its phenolic hydroxyl groups, which donate electrons to stabilize free radicals. Modifying ammonium lignosulfonate by chemical treatment under DBD plasma can increase the exposure or concentration of these active phenolic sites. The obtained results of the DPPH assay evidenced the good antioxidant capacity of all investigated MLSs, where the IC_50_ values ranged between 8.08 and 11.7 mg/mL. We found that the antioxidant activity of the modified lignosulfonates (MLS) was not affected after chemical modification, being observed a slight decrease in IC_50_ comparing to the ALS value presented in Figure 6, where the ALS-BL sample evidenced the most decreased value of IC_50_.

A direct comparison of the antioxidant activity of the investigated lignosulfonates with Trolox standard through Trolox equivalent antioxidant capacity (TEAC) expression provides an independent assessment of the antioxidant capacity of a compound. The modified lignosulfonates examined in this study had TEAC values in the range of 0.0248 and 0.0359, which were higher than ALS value, indicating an increased antioxidant capacity (Table 6).

## 4. Conclusions

A new method for the preparation of the multifunctional lignosulfonate-based microparticles with good homogeneity, functionalized surface and improved thermal properties has been developed. It consists of chemical treatment of the ammonium lignosulfonate with carboxylic acids such as lactic acid, oleic acid or γ-butyrolactone under DBD plasma conditions which mainly assure surface modification of the microparticles. Utilization of lignins in various applications requires a proper detailed investigation of their physico-chemical characteristics to understand their chemical nature and physical behavior. This study may contribute to the development of multifunctional lignin-based materials with improved thermal and surface properties. Water-soluble MLS microparticles can assemble leading to larger spherical aggregates with different sizes that have a homogeneous polydispersity in respect with ALS. The tendency to agglomerate is due to the stronger cohesion forces or due to the H-bonds established between the hydroxyl groups of lignosulfonate derivatives. Some modified products showed the presence of disaggregated domains containing cavities formed by the release of the lower lignosulfonate particle aggregates that exhibited a large specific surface area, which can improve compatibility with other materials that are very important in applications of the lignin-based materials in food packaging and safety as well as biomedical fields. These differences are crucial in using lignin because they affect the thermal stability, compatibility and the solubility parameters. The TG/DTG/DTA and DSC results evidenced the improved thermal stability of the microparticles of the MLS. These results demonstrate that MLSs microparticles have very interesting properties; hence, they could be potential materials in pharmaceutical, food and cosmetic applications due to their hierarchical structures and designable multi applications.

## Figures and Tables

**Table 1:** Average weight molecular weight (Mw) and second virial coefficient (A_2_) for modified lignosulfonates.

Sample	Mw (kDa)	A_2_ × 10^−3^ (mL mol g^−2^)
ALS	167	2.45
ALS-LA	325	1.51
ALS-OA	969	2.50
ALS-BL	1385	2.76

**Table 2:** Particle size analysis data of the studied samples.

Sample	Z-Average, d (nm)	PDI
ALS	245; 800	1.00
ALS-LA	370	0.74
ALS-OA	400	0.72
ALS-BL	277	0.69

**Table 3:** Thermogravimetric data of unmodified and modified lignosulfonates under plasma treatment.

Sample	Peak I			Peak II			Peak III			Mass Loss at 600 °C (wt%)
	T_max I_ (°C)	Mass Loss (wt%)	Mass Loss Rate (wt%/min)	T_max II_ (°C)	Mass Loss (wt%)	Mass Loss Rate (wt%/min)	T_max III_ (°C)	Mass Loss (wt%)	Mass Loss Rate (wt%/min)	
ALS	70.4	3.3	−0.65	249.4	33.6	−3.23				90.3
ALS-LA	91.8	2.9	−0.29	243.5	23.3	−3.42	364.8	46.6	−1.48	72.8
ALS-OA	93.9	2.5	−0.55	253.3	21.5	−3.12	364.7	41.2	−1.61	65.2
ALS-BL	96.0	4.4	−0.50	251.3	23.2	−3.10				61.4

T_max I_; T_max II_ and T_max III_—temperature corresponding to the maximum of the peak in DTG curves of the lignosulfonates.

**Table 4:** Thermal characteristics obtained from DSC curves (first heating run) for unmodified and modified lignosulphonates.

Sample	T_g_ (°C)	ΔC_p_ (J/g·°C)
ALS	92.36	-
ALS-LA	61.44; 96.90;	-
ALS-OA	81.70	0.250
ALS-BL	74.33	0.233

**Table 5:** RSA of the studied lignosulfonates, at concentration of 10 mg/mL.

Sample	Mean RSA %
ALS	49.6 ± 2.97
ALS-LA	45.1 ± 1.84
ALS-OA	46.1 ± 3.11
ALS-BL	47.65 ± 2.76

**Table 6:** Trolox equivalent antioxidant capacity (TEAC) values of the studied lignosulfonates.

Sample	TEAC [mg Trolox/mg Samples]
ALS	0.0241
ALS-LA	0.0248
ALS-OA	0.0355
ALS-BL	0.0359

## Data Availability

The original contributions presented in this study are included in the article. Further inquiries can be directed to the corresponding author.
